# Genome-Wide and Gene-Based Association Studies of Anxiety Disorders in European and African American Samples

**DOI:** 10.1371/journal.pone.0112559

**Published:** 2014-11-12

**Authors:** Takeshi Otowa, Brion S. Maher, Steven H. Aggen, Joseph L. McClay, Edwin J. van den Oord, John M. Hettema

**Affiliations:** 1 Department of Psychiatry, Virginia Institute for Psychiatric and Behavioral Genetics, Virginia Commonwealth University, Richmond, Virginia, United States of America; 2 Department of Neuropsychiatry, Graduate School of Medicine, University of Tokyo, Tokyo, Japan; 3 Department of Mental Health, Johns Hopkins Bloomberg School of Public Health, Baltimore, Maryland, United States of America; 4 Department of Pharmacy, Center for Biomarker Research and Personalized Medicine, Virginia Commonwealth University, Richmond, Virginia, United States of America; University Of São Paulo, Brazil

## Abstract

Anxiety disorders (ADs) are common mental disorders caused by a combination of genetic and environmental factors. Since ADs are highly comorbid with each other, partially due to shared genetic basis, studying AD phenotypes in a coordinated manner may be a powerful strategy for identifying potential genetic loci for ADs. To detect these loci, we performed genome-wide association studies (GWAS) of ADs. In addition, as a complementary approach to single-locus analysis, we also conducted gene- and pathway-based analyses. GWAS data were derived from the control sample of the Molecular Genetics of Schizophrenia (MGS) project (2,540 European American and 849 African American subjects) genotyped on the Affymetrix GeneChip 6.0 array. We applied two phenotypic approaches: (1) categorical case-control comparisons (CC) based upon psychiatric diagnoses, and (2) quantitative phenotypic factor scores (FS) derived from a multivariate analysis combining information across the clinical phenotypes. Linear and logistic models were used to analyse the association with ADs using FS and CC traits, respectively. At the single locus level, no genome-wide significant association was found. A trans-population gene-based meta-analysis across both ethnic subsamples using FS identified three genes (*MFAP3L* on 4q32.3, *NDUFAB1* and *PALB2* on 16p12) with genome-wide significance (false discovery rate (FDR] <5%). At the pathway level, several terms such as transcription regulation, cytokine binding, and developmental process were significantly enriched in ADs (FDR <5%). Our approaches studying ADs as quantitative traits and utilizing the full GWAS data may be useful in identifying susceptibility genes and pathways for ADs.

## Introduction

Anxiety disorders (ADs) are common mental disorders characterized by excessive, prolonged, and debilitating levels of anxiety, with substantial lifetime prevalence [1]. They are subdivided into clinical diagnostic categories such as generalized anxiety disorder (GAD), panic disorder (PD), posttraumatic stress disorder (PTSD), obsessive-compulsive disorder (OCD) and phobias, based on their onset, symptoms, and course. ADs are complex diseases that are caused by a combination of genetic and environmental factors. Family and twin studies have demonstrated that ADs have significant familial aggregation, and their heritability estimates range from 30 to 50% [2], [3].

Numerous genetic studies of ADs have been conducted targeting candidate genes. The most intensively studied candidate genes are related to neurotransmitter systems involved in the regulation of anxiety, neuropeptides, and stress response [3]. However, most of these studies have produced inconsistent or negative results. One of the reasons for inconsistency between studies may be due to Type I error from poorly-chosen candidates or Type II error due to small sample size underpowered to detect individual susceptibility variants of small effect.

Genome-wide association studies (GWAS) have proven to be a successful method for the identification of common genetic variants that increase susceptibility to complex diseases or traits. Recently, several GWAS of ADs such as PD [4], [5], PTSD [6], [7], [8], OCD [9], and phobias [10] have been published. However, the top findings in these studies have not overlapped with previous candidates and explained only small proportions of the total genetic variance. The failure to replicate the same single nucleotide polymorphisms (SNP) between studies may be attributable to poor power with small sample sizes and allelic and/or phenotypic heterogeneity across populations.

GWAS usually focus on the most significant individual variants without considering the global evidence of the gene tested. Unlike genetic variants that have different allele frequencies, linkage disequilibrium (LD) structure, and heterogeneity across diverse human populations, the gene itself is highly consistent across populations [11]. Gene-based analysis might produce more consistent results and improve power with a smaller number of statistical tests. Furthermore, with the gene as the unit of analysis, biological pathway analysis using available functional information might facilitate the identification of the pathogenic mechanisms of complex diseases such as ADs. Therefore, both gene- and pathway-based analyses could have better statistical power for detecting susceptibility loci and provide a complementary approach to single-locus analysis.

Most genetic association studies focus on categorical traits comparing allele frequencies for diagnosed cases versus controls. However, if GWAS indicate that multiple genes affect these disorders, this implies that their genetic liability is distributed quantitatively rather than qualitatively [12]. For common disorders like ADs, disease states can be interpreted as being the extremes of continuous liability dimensions. Therefore, statistical power can be enhanced by studying the broader distribution than by dichotomizing the same distribution into cases and controls [12], [13]. Furthermore, previous studies suggest that ADs exhibit strong lifetime comorbidity, partially due to shared genetic risk factors between them and with anxious personality traits like neuroticism [14], [15], [16]. Therefore, studying AD phenotypes in a coordinated manner is an alternative approach to increase the statistical power for identifying susceptibility genes for ADs, as demonstrated by prior reports from our group [17].

The aim of this study is to conduct GWAS of ADs as quantitative traits as well as categorical traits in unselected population samples from the United States consisting of 2540 European American (EA) and 849 African American (AA) subjects. To optimally utilize the GWAS data sets, we examined our results at 3 levels: (i) SNP-based analyses (ii) gene-based analyses and meta-analyses combining these results, and (iii) gene-set based analyses.

## Materials and Methods

### Subjects

Data for the analyses came from the “control” sample originally part of a large schizophrenia study (Molecular Genetics of Schizophrenia (MGS)). The full MGS control sample is described in detail elsewhere [18]. Briefly, the available sample consisted of unrelated subjects selected during 2004–2007 by random digit dialing from approximately 60,000 US households. Institutional review board approval was obtained at NorthShore University HealthSystem. Participants first consented online to use of their DNA and phenotypic information for the study of any illness or trait and then signed an identical hard-copy consent at the time of venipuncture. They were screened and excluded for psychotic and bipolar disorders for use as a comparison group for genetic association studies of these more severe psychiatric phenotypes but were not excluded for other common psychiatric disorders such as depression and anxiety. Self-reported ancestry [19] was confirmed by genotypic data with ancestry-informative markers [18]. The data were obtained with permission from dbGaP (Database of Genotypes and Phenotypes, http://www.ncbi.nlm.nih.gov/gap, Study Accessions: phs000021.v3.p2 (“Genetic Association Information Network (GAIN)”) and phs000167.v1.p1 “nonGAIN”). Data for the EA subjects were combined from both the GAIN (n = 1442) and nonGAIN (n = 1367) datasets. These derived from the same original sample but had been separately deposited into dbGaP. Data for the AA subjects were obtained from the GAIN subsample (n = 979).

### Diagnostic measures

All MGS control subjects completed an online psychiatric screening interview that included the lifetime version of the Composite International Diagnostic Interview, Short Form (CIDI-SF) [20]. The CIDI-SF is accurate compared with the full CIDI [20] and has been used for self-report. Because of its brevity and cost effectiveness, the CIDI-SF is suitable for an online interview to screen common psychiatric disorders in the general population [18]. For those subjects with requisite response data, we applied DSM-based algorithms to the CIDI-SF responses to obtain the following six lifetime clinical phenotypes: major depression (lifetime prevalence of total sample, 30.0%), GAD (18.4%), panic attacks (2.1%), agoraphobia (6.5%), social phobia (14.3%), and specific phobia (11.7%). We note that only panic attacks, and not panic disorder, could be identified due to limitations in the items included in that section of the CIDI-SF. In the present study, we used the latter five AD phenotypes for the analyses. Besides attempting to identify subjects meeting full symptomatic criteria (“cases”, score  = 2), we also sought to differentiate subjects who were highly symptomatic but did not meet full criteria (“sub-syndromal”, score  = 1) versus those with few or no reported symptoms (“unaffecteds”, score  = 0). This was operationalized by either (i) keeping the full symptomatic criteria and removing the diagnostic requirements of distress/impairment or (ii) reducing the symptomatic severity or duration. This strategy produced ordered, rather than classification variables that served as input indicators for the factor analyses described below. It also identifies more extreme comparison groups for use in case-control (CC) analyses, since diagnostic thresholds are defined for clinical purposes and may not sufficiently differentiate subjects by the risk alleles they carry.

Given prior evidence supporting shared genetic liability across these AD phenotypes [14], [15], [16], we performed factor analyses to estimate an overall score (factor score; FS) for each subject. Due to substantial correlation between phenotypic and genetic factor structure of ADs, this approach should provide FS that represent shared genetic risk. We conducted the analyses as reported in our previous paper [17]. Briefly, we entered scores for the five AD clinical phenotypes into factor analyses in Mplus (version 4) [21]. Exploratory factor analyses with one versus two latent factors each produced reasonable solutions that adequately fit the data, so, we chose to use the former solution representing a single common factor. The overall factor structure was similar across the AA and EA subjects although their thresholds somewhat differed, indicating differences in frequency of phenotypic scores (prevalence) between populations. We constrained factor loadings to be equal across these two samples in order to score all subjects in a manner that was consistent across populations. A confirmatory factor analysis was carried out in Mplus to estimate a single FS for each subject for use as quantitative phenotype in association analyses.

### Statistical analyses

To correct for multiple testing, false discovery rate (FDR, *q*-value) was calculated, which is an estimate of the proportion of false discoveries among all significant markers when the corresponding *p*-value is used as the threshold for declaring significance [22]. This approach provides a good way to find true effects controlling false discoveries and is much less affected by number of tests, which is an arbitrary factor [23]. *Q*-values less than 5% and 25%, respectively, were taken as significant and suggestively significant for SNP-, gene-, and pathway-based analyses.

#### SNP-based analysis

As previously described in detail [24], DNA samples were genotyped at the Broad Institute using the Affymetrix 6.0 array. Quality control procedures excluded about 5% of all subjects due to low genotype call rates (<0.95), heterozygosity outliers, sample duplicates, sex typing discrepancies, or genetic relatedness. SNPs were excluded if a SNP call rate <0.95, Hardy-Weinberg equilibrium *p*-value <1×10^−5^, and minor allele frequency (MAF) <0.05 for both cases and controls. A total of 626,833 (EA) and 730,090 (AA) autosomal SNPs were available for further analyses.

We performed regression analyses assuming additive genetic effects in PLINK [25] to test the main effects of SNPs on the outcome phenotypes. These included gender and age as covariates, given their strong association with ADs. To account for the genetic substructure of human populations, multidimensional scaling (MDS) was used. MDS produced eight components, none of which were correlated with FS or CC status. Therefore, no MDS dimensions were included as covariates in the analyses. In the present study, two phenotypic strategies were compared. First, linear regression analyses including all subjects were conducted using FS as a quantitative outcome variable. These scores incorporate all of the phenotypic information in a statistically coordinated fashion from the factor analyses. Second, logistic regression was applied in a CC approach, designating subjects scoring a “2” for any of the clinical phenotypes as a “case” versus those scoring “0” on all as “hyper-normal” controls (no full or subsyndromal AD or major depression).

Quantile-quantile (QQ) plots were used to evaluate overall significance of the GWA analyses and the potential impact of population stratification. The inflation factor λ was calculated on the basis of the median chi-square. Haploview 4.2 [26] was used to create Manhattan plots of *p*-values from the GWA analyses and to examine LD between markers. Power calculations were performed using the program Quanto v1.2.4 (http://hydra.usc.edu/gxe).

#### Gene-based analysis

Gene-based association analysis in each population was performed using the versatile gene-based test for genome-wide association studies (VEGAS) [27]. In brief, VEGAS tests for association on a per-gene basis, by considering the *p*-value of all SNPs within genes (including +/−50 kb from the 5′ and 3′ UTR), accounting for LD and number of SNPs per gene. For a given gene with n SNPs, association *p*-values were first converted to upper tail chi-squared statistics with 1 degree of freedom (df). The observed gene-based test statistic was then the sum of all of the chi-squared 1 df statistics within the gene. Using the Monte Carlo simulation, the empirical gene-based *p*-value was calculated as the proportion of simulated test statistics that exceeded the observed gene-based test statistic.

Previous studies have found consistent genetic effects on common diseases across different racial groups even if LD patterns and allele frequencies differ considerably across populations [11], [28]–[30]. Therefore, to increase statistical power, trans-population meta-analysis of gene-based GWA analyses using FS or CC was conducted. To test overall significance, Stouffer's Z-score method [31] implemented in METAL [32] was used. Z-scores for each gene were combined across samples in a weighted sum, with weights proportional to the square-root of the sample size for each study [31]. Given unequal numbers of cases and controls, the effective sample sizes were calculated as N_eff_  = 4/(1/N_cases_+1/N_controls_) for the CC analyses.

#### Gene-set enrichment analysis

Gene-set enrichment analysis was carried out to complement the results from gene-based GWA analyses and to determine which potential biological pathways could play a role in ADs. For the analysis, we included all genes from the gene-based meta-analysis test with a *p*-value <0.01 using the public domain tool provided by the Database for Annotation, Visualization and Integrated Discovery (DAVID) bioinformatics platform [33]. We used gene ontology (GO) to create gene-sets because it provided the largest amount of information and is well structured. Considering the redundant nature of annotations, groups of similar annotations were combined using ‘Functional Annotation Clustering’ (kappa value>0.5). We selected the best significantly enriched terms of individual groups. By performing these enrichment analyses, we attempted to identify whether the genes most associated with ADs were more prevalent in any known GO terms than would be expected by chance.

## Results

### SNP-based analysis

After application of QC parameters, 2540 EA and 849 AA subjects had FS values available for analyses. Of these, samples for case-control analysis consisted of 1697 EA subjects (757 cases and 940 controls) and 597 AA subjects (324 cases and 273 controls), respectively (Table 1). The genomic inflation factors λ for the FS and CC analyses, respectively, were 1.004 and 1.005 in EA and 1.004 and 1.008 in AA, suggesting no significant inflation (Figure S1 in File S1). The QQ and Manhattan plots for theses SNP-based GWA analyses are displayed in Figures S1 and S2 in File S1.

**Table 1:** Demographic characteristics of European and African American samples.

Sample	European American		African American	
	case	control	case	control
Factor score	2540		849	
Gender ratio (female/male)	1.07		1.60	
Age (s.d.)	50.8 (16.4)		45.6 (13.3)	
Case control	757	940	324	273
Gender ratio (female/male)	1.72	0.67	2.34	1.05
Age (s.d.)	48.3 (14.6)	52.1 (17.2)	44.4 (12.6)	46.2 (13.3)

s.d., standard deviation.

Overall, no SNP reached genome-wide significance or suggestive significance for any GWA analyses in either the EA or AA samples (Table 2 and Figure S2 in File S1). The most significant signal was observed at SNP rs4692589 located in *MFAP3L* on 4q32.3 from the results using the FS traits in the EA sample (*p* = 8.63×10^−7^, *q* = 0.37; Table 2). Of note, among top findings in the same analysis was SNP rs2170820, located in *TMEM132D* (12q24.3), a gene which has been reported to be associated with PD in a European Caucasian sample [4].

**Table 2:** Top findings of SNP-based GWAS in the European (EA) and African American (AA) samples.

Sample	SNP	Chr	Position (bp)	A1	Beta (OR)*	*p*	*q*	Gene
EA	FS							
	rs4692589	4	171,171,820	C	−0.067	8.63E-07	0.37	*MFAP3L*
	rs10893268	11	123,943,822	T	−0.069	1.18E-06	0.37	*OR8A1*
	rs12153327	5	100,729,499	C	−0.064	2.02E-06	0.42	
	rs7657455	4	171,195,418	G	−0.064	2.90E-06	0.45	*MFAP3L*
	rs10016872	4	171,116,951	C	−0.061	3.97E-06	0.48	
	rs2170820	12	128,588,656	C	−0.063	4.63E-06	0.48	*TMEM132D*
	rs1551277	7	47,795,842	A	0.068	6.88E-06	0.53	*PKD1L1*
	rs12703441	7	141,539,176	C	−0.062	6.97E-06	0.53	
	rs6463447	7	47,797,795	A	0.068	7.60E-06	0.53	*PKD1L1*
	rs4736192	8	140,049,007	A	0.073	9.24E-06	0.53	
	CC							
	rs2115691	4	74,962,555	G	0.67	4.45E-06	0.90	*CXCL1*
	rs3097411	4	74,957,318	C	0.68	6.58E-06	0.90	*CXCL1*
AA	FS							
	rs7141336	14	90,355,981	C	0.19	5.83E-06	1.00	*TTC7B*
	rs17548918	3	21,355,095	C	0.26	8.75E-06	1.00	
	rs241257	1	4,514,682	A	0.17	9.09E-06	1.00	
	CC							
	rs12120353	1	4,520,856	A	2.13	3.08E-06	0.99	
	rs10762651	10	76,492,123	T	0.54	6.06E-06	0.99	*DUPD1*
	rs17105932	11	106,048,675	T	0.45	6.91E-06	0.99	*GUCY1A2*
	rs6799682	3	178,659,210	A	1.79	7.53E-06	0.99	
	rs17105964	11	106,074,793	G	0.46	8.74E-06	0.99	*GUCY1A2*

GWAS, genome-wide association study; SNP, single nucleotide polymorphism.

Chr, chromosome; bp, base position; OR, odds ratio; FS, factor score analysis; CC, case-control analysis.

A1, minor allele based on whole sample.

*Beta was calculated with FS and OR was calculated with CC.

Genes with SNPs located up to 20 kb down- or upstream were shown.

SNPs with *p*-values <10^−5^ were shown.

### Gene-based analysis

Using VEGAS, SNPs in each GWA analysis were mapped to approximately 17,700 genes (FS-EA: 17,660, FS-AA: 17,678, CC-EA: 17,655, CC-AA: 17,669). No deviations from the expected distribution of *p*-values were observed in the QQ plots of each gene-based analysis (Figure S3 in File S1). Using FS, a gene reached a significant *q*-value (*MFAP3L*, *q* = 0.035; Table 3) and another 11 genes reached suggestive significance (*q*<0.25) in the EA sample, whereas none reached suggestive significance in the AA sample. Using CC, three genes reached suggestive significance (*PF4V1*, *CXCL1*, and *CXCL6*) in the EA sample, whereas none reached suggestive significance in the AA sample.

**Table 3:** Meta-analysis of gene-based GWA analyses in European-American (EA) and African-American (AA) samples.

				Meta-analysis		EA			AA		
Gene	Chr	Start (bp)	Stop (bp)	*p*	*q*	nSNPs	*p*	*q*	nSNPs	*p*	*q*
FS											
*NDUFAB1*	16	23,499,835	23,515,140	4.04E-06	0.028	14	9.00E-06	0.053	16	1.26E-01	0.98
*PALB2*	16	23,521,983	23,560,179	4.37E-06	0.028	17	6.00E-06	0.053	23	1.78E-01	0.98
*MFAP3L*	4	171,144,322	171,184,004	4.78E-06	0.028	40	2.00E-06	0.035	43	3.59E-01	0.98
*AADAT*	4	171,217,947	171,247,947	1.64E-05	0.072	27	3.40E-05	0.12	27	1.50E-01	0.98
*UBFD1*	16	23,476,362	23,493,211	3.66E-05	0.13	17	2.00E-05	0.09	18	3.84E-01	0.98
*EARS2*	16	23,440,834	23,476,197	4.41E-05	0.13	22	4.10E-05	0.12	25	2.86E-01	0.98
*DCTN5*	16	23,560,307	23,588,683	5.71E-05	0.14	17	1.13E-04	0.20	22	1.73E-01	0.98
*CTNNBL1*	20	35,755,847	35,933,934	6.80E-05	0.15	76	1.05E-03	0.68	83	2.21E-02	0.98
*VSTM2L*	20	35,964,912	36,007,161	9.20E-05	0.16	39	6.07E-04	0.54	40	5.97E-02	0.98
*GGA2*	16	23,383,143	23,429,309	9.89E-05	0.16	19	9.60E-05	0.19	22	3.02E-01	0.98
*C11orf40*	11	4,549,228	4,555,626	1.03E-04	0.16	54	8.30E-05	0.18	59	3.41E-01	0.98
*COG7*	16	23,307,316	23,372,004	1.32E-04	0.19	29	7.10E-05	0.18	32	4.44E-01	0.98
*SRXN1*	20	575,267	581,890	1.60E-04	0.22	42	4.79E-03	0.83	47	7.76E-03	0.98
*TARS*	5	33,476,654	33,503,953	2.27E-04	0.29	17	1.63E-04	0.25	25	3.98E-01	0.98
*OR52I2*	11	4,564,618	4,565,671	2.49E-04	0.29	51	1.68E-04	0.25	56	4.17E-01	0.98
CC											
*PF4V1*	4	74,937,876	74,939,062	6.64E-05	0.37	29	4.00E-06	0.059	44	9.61E-01	0.99
*CXCL1*	4	74,953,972	74,955,817	8.51E-05	0.37	32	7.00E-06	0.059	48	8.96E-01	0.98
*CXCL6*	4	74,921,276	74,923,341	1.27E-04	0.37	19	1.00E-05	0.059	30	9.45E-01	0.99

Genes with *q*-values <5% or <25% in either EA, AA, or meta-analysis of the two populations were shown.

GWA, genome-wide association; Chr, chromosome; bp, base position; nSNPs, number of SNPs located within the gene.

FS, factor score analysis; CC, case-control analysis.

Although there was no full overlap of top associated genes between the two populations, several genes showed evidence of association in both. Therefore, we conducted trans-population meta-analyses of gene-based studies using FS or CC. Top findings from the gene-based meta-analysis using FS and CC are shown in Table 3. Three genes met the criteria for genome-wide significance according to the threshold that allows for 5% false discovery rate (*NDUFAB1*, *PALB2*, and *MFAP3L*; all *q*-values  = 0.028). Ten other genes in five loci reached a suggestively significant level using FS (4q32, 11p15, 16p12, 20p13, and 20q11), while three genes reached the genome-wide suggestive level using CC (*PF4V1*, *CXCL1*, and *CXCL6*; Table 3).

### Gene-set enrichment analysis

We next examined all genes with *p*<0.01 in the gene-based meta-analysis to see whether they were enriched with known GO terms, using DAVID with the whole genome background as a base set. The numbers of genes included in the analyses were 296 (FS) and 322 (CC). GO enrichment analysis showed that two terms using FS (“pattern specification process” and “cytokine binding”) and two terms using CC (“nucleoplasm” and “transcription regulator activity”) were significantly enriched in ADs (*q*<0.05) (Table 4).

**Table 4:** GO term enrichment analysis based on the results from the meta-analysis of gene-based GWAS in European and African American samples.

GO term		Count	%	Fold Enrichment	*p*	*q*
FS						
GO: 0007389	B: pattern specification process	14	5.0	3.6	0.0002	0.0025
GO: 0019955	M: cytokine binding	9	3.2	5.5	0.0002	0.0030
GO: 0000793	C: condensed chromosome	7	2.5	3.6	0.0132	0.16
GO: 0030155	B: regulation of cell adhesion	7	2.5	3.5	0.0154	0.23
GO: 0016563	M: transcription activator activity	13	4.6	2.1	0.0196	0.24
CC						
GO: 0005654	C: nucleoplasm	26	9.2	2.0	0.0015	0.019
GO: 0030528	M: transcription regulator activity	40	14.1	1.6	0.0017	0.024
GO: 0009952	B: anterior/posterior pattern formation	8	2.8	3.8	0.0049	0.078

GWAS, genome-wide association study; FS, factor score analysis; CC, case-control analysis.

B, biological process; M, molecular function; C, cellular component.

## Discussion

We report here the results from the GWAS of ADs in two populations using quantitative and categorical phenotypes at SNP, gene, and pathway levels. We included 2540 EA and 849 AA subjects from the MGS control sample and conducted association analyses using two phenotypic approaches that sought to combine information across these disorders based upon prior research that suggests that they possess shared genetic risk factors. The first utilized factor analysis to extract a single phenotypic score for each subject for use as a quantitative trait. The second approach focused on categorical diagnoses that compared allele frequencies for clinical cases versus hyper-normal controls.

There were notable differences between results obtained using the two phenotypic methods. Most of the top SNPs were not the same, although many that were nominally significant in one were also in the other. The overall significance of SNPs (*p*- and *q*-values) was greater using FS than CC in the EA sample. This is not surprising, as the factor analytic phenotypes should provide more powerful targets for genetic association than the categorical phenotypes for several reasons: quantitative traits generally have greater information content, and there were more subjects with useable quantitative traits than categorical traits [34]. However, this was not the case with the AA sample where the significance of top findings was similar between the FS and CC analyses (Tables 2). In the AA sample, the difference in the sample sizes between the FS and CC analyses was not as large as for the EA sample, which may result in the smaller differences in power.

At the SNP level, none of the SNP results reached genome-wide significance. The most likely reasons for the modest *p*-values seen in each GWA analysis may be insufficient power to detect very small genetic effects. In the FS analyses, the power of our samples were 35% in EA (n = 2540) and 0.6% in AA (n = 849) with an additive model, a type I error rate of 5×10^−8^, and an effect size explaining of 1% total variance. In the CC analyses, the power of the samples were 0.09% in EA (757 cases vs. 940 controls) and 0.01% (324 cases vs. 273 controls) with the log-additive model, a type I error rate of 5×10^−8^, a frequency of 0.25 and an effect size of 1.2. Therefore, it was not surprising that we could not detect any locus of genome-wide significance.

While SNP-based GWA analysis focuses on the most significant individual variants, the gene-based approach tests the global null hypothesis about the SNPs located per gene. Gene-based tests allowed us to explore the impact of multiple variants in a gene even if the gene did not contain any SNP reaching genome-wide significance. Therefore, we performed gene-based GWA analyses in the present study. Only when using FS in the trans-population gene-based meta-analysis did we detect significant association signals in three genes (*MFAP3L* on 4q32.3 and *NDUFAB1* and *PALB2* on 16p12). We will review these genes in turn.

NDUFAB1 is a subunit of NADH dehydrogenase, a nuclear encoded subunit of mitochondrial Complex I, which regulates the redox status of nicotinamide adenine dinucleotide/nicotinamide adenine dinucleotide hydride (NAD/NADH), and could be observed in both the cytoplasm and nucleus. NDUFAB1 may be involved in the regulation of NAD and NADH which influence fundamental cellular processes such as cellular metabolism, gene expression, and ion channel regulation, although the function of NDUFAB1 in ADs remains to be established [35], [36].

*PALB2* encodes for the protein PALB2, which co-localizes with BRCA2 in the cell nucleus and promotes its localization and stability in cellular structure like chromatin and nuclear matrix [37]. SNP rs420256 in *PALB2* has been reported to be associated with bipolar disorder in Caucasians in previous studies [38], [39]. In our study, rs420259 did not show any significant association with ADs in either the EA or AA samples (FS-EA *p* = 0.11; FS-AA *p* = 0.37). Rs8062954 on the same LD block (r^2^ = 0.12, D' = 1.00) with rs420259 was nominally associated with ADs using FS in the AA sample (FS-AA *p* = 4.88×10^−5^).

*MFAP3L* encodes a transmembrane protein, microfibrillar-associated protein 3-like. The intracellular region of this protein reportedly contains a cluster of phosphorylation sites and phosphatidylinositol-3 kinase (PI3K) regulatory subunit, which suggests the involvement of MFAP3L in the signal transduction of PI3K/AKT pathway [40]. The PI3K/AKT pathways are known for regulating metabolism, cell growth, and cell survival [41]. Recent studies have indicated that both dopamine and serotonin partially exert their actions by modulating the activity of AKT [42], [43]. Furthermore, chromosome 4q32 region was suggested to be associated with ADs (PD, SAD, and phobia) in a previous linkage study [44], although this region was not confirmed in a recent meta-analysis of linkage data for ADs [45].

To further explore the GWAS data, we took a pathway-based approach, providing complementary information to single-marker and gene-based methods. In the GO term enrichment analyses, we found two terms (“pattern specification process” and “cytokine binding”) using FS and two terms (“nucleoplasm” and “transcription regulator activity”) using CC to be significantly enriched in ADs, based on the results from the trans-population gene-based meta-analysis. As observed in the SNP- and gene-based analyses, the significance of the top pathways was greater using FS than CC (Table 4). Among these significant pathways, one intriguing pathway is “cytokine binding”, proteins of which function to control the survival, growth and differentiation of tissues and cells via interaction with cytokines. Recent studies have suggested that inflammation of neurons and inflammatory cytokine production contribute to the pathophysiology of depression and anxiety [46]. Actually, cytokines and their signaling pathways have significant effects on the metabolism of multiple neurotransmitters such as serotonin, dopamine, and glutamate and on synaptic plasticity [47]. An increase in inflammatory markers such as IL-1, IL-6, TNF-alpha, and IFN-gamma have been documented in ADs including PTSD, PD, and OCD as well as anxiety-related personality traits such as neuroticism [46]. The genes in the significant pathways are worthy of follow-up in the future research.

Several limitations in this study should be addressed. First, in the present study, we did not perform replication analyses of the top findings identified in the SNP-, gene-, pathway-based analyses. However, to increase the statistical power, we conducted the meta-analyses of the gene-based analyses by combining the two populations. In future research, further replication studies are needed to confirm our findings. Second, the distribution of FS used for the GWA analyses was not normally distributed. Like most psychiatric phenotypes, the distribution was quite skewed, with many of the unaffected subjects falling under a peak at the lower end of the score. Therefore, *p*-values for some markers in the GWAS might have been biased by this feature of the FS distribution. We tested this for SNPs on different chromosomes by comparing normal-theory regression with permutation testing and did not detect major differences. Third, the gender ratio differs significantly between cases and controls in both EA and AA samples, which may affect results. Therefore, we conducted the analyses controlling for gender and age instead of conducting gender-specific analyses because it reduce the statistical power. Fourth, the gene- and pathway-based analyses assumed that the local SNPs only modify the function of the local gene. Thus, both *cis* and *trans* regulation of the genes should be considered in the future analyses [48]. Fifth, since the sample size of the EA sample was much larger than the AA sample, most of the top findings in the gene-based meta-analysis were found in the EA sample. Therefore, caution is needed to interpret the results given genetic heterogeneity between the two populations.

In conclusion, our results demonstrate the potential advantage of studying AD phenotypes as quantitative traits for identifying shared susceptibility genes. In addition, our study provides a strategy to utilize the full information of GWAS to find new genes and pathways that would be missed in a single SNP analysis. Further studies are necessary to confirm our findings and clarify the underlying mechanisms of ADs.

## Supporting Information

File S1 **Figure S1 in File S1 Quantile-quantile (QQ) plots of each SNP-based genome-wide association analysis.** (*a*) FS-EA, (*b*) FS-AA, (*c*) CC-EA, (*d*) CC-AA. FS, factor score analysis; CC, case-control analysis; EA, European Americans; AA, African Americans. **Figure S2 in File S1 Manhattan plots of each genome-wide association analysis.** (a) FS-EA, (b) FS-AA, (c) CC-EA, (d) CC-AA. FS, factor score analysis; CC, case-control analysis; EA, European Americans; AA, African Americans. **Figure S3 in File S1 Quantile-quantile (QQ) plots of gene-based genome-wide association analysis.** (a) FS-EA, (b) FS-AA, (c) CC-EA, (d) CC-AA. FS, factor score analysis; CC, case-control analysis; EA, European Americans; AA, African Americans.
(DOCX)
