# Supplementary material for: Genome-Wide and Gene-Based Association Studies of Anxiety Disorders in European and African American Samples
Source: PLoS One. 2014 Nov 12;9(11):e112559. doi: 10.1371/journal.pone.0112559 (PMC4229211; doi:10.1371/journal.pone.0112559)
Supplement: File S1 — Figure S1 in File S1 Quantile-quantile (QQ) plots of each SNP-based genome-wide association analysis. (a) FS-EA, (b) FS-AA, (c) CC-EA, (d) CC-AA. FS, factor score analysis; CC, case-control analysis; EA, European Americans; AA, African Americans. Figure S2 in File S1 Manhattan plots of each genome-wide association analysis. (a) FS-EA, (b) FS-AA, (c) CC-EA, (d) CC-AA. FS, factor score analysis; CC, case-control analysis; EA, European Americans; AA, African Americans. Figure S3 in File S1 Quantile-quantile (QQ) plots of gene-based genome-wide association analysis. (a) FS-EA, (b) FS-AA, (c) CC-EA, (d) CC-AA. FS, factor score analysis; CC, case-control analysis; EA, European Americans; AA, African Americans. (DOCX) [file pone.0112559.s001.docx]

**File S1**


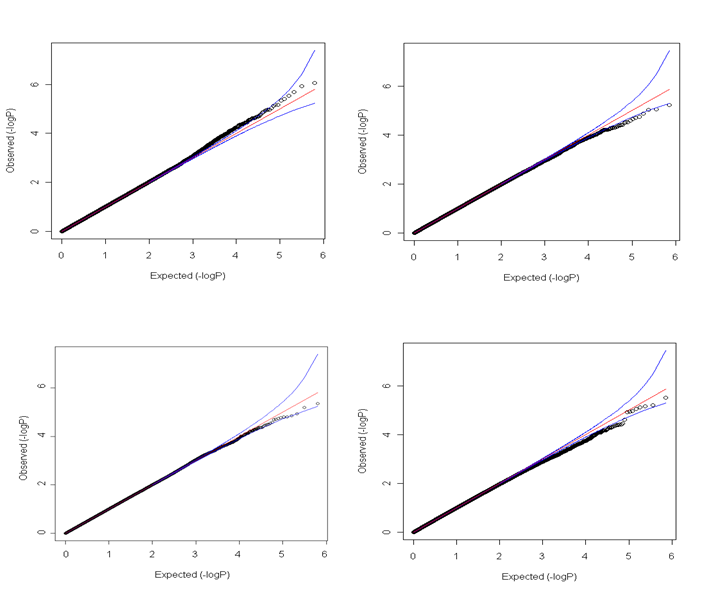

Expected (-logP)

Expected (-logP)

Expected (-logP)

Expected (-logP)

**(a)**

**(d)**

**(c)**

**(b)**

**Figure S1 Quantile-quantile (QQ) plots of each SNP-based genome-wide association analysis.** (*a*) FS-EA, (*b*) FS-AA, (*c*) CC-EA , (*d*) CC-AA. FS, factor score analysis; CC, case-control analysis; EA, European Americans; AA, African Americans. The observed –log_10_(*p*-value) are plotted against the expected –log_10_(*p*-value) under no association (straight red line) The 95% confidence interval of expected values is indicated in each figure (upper and lower blue line).


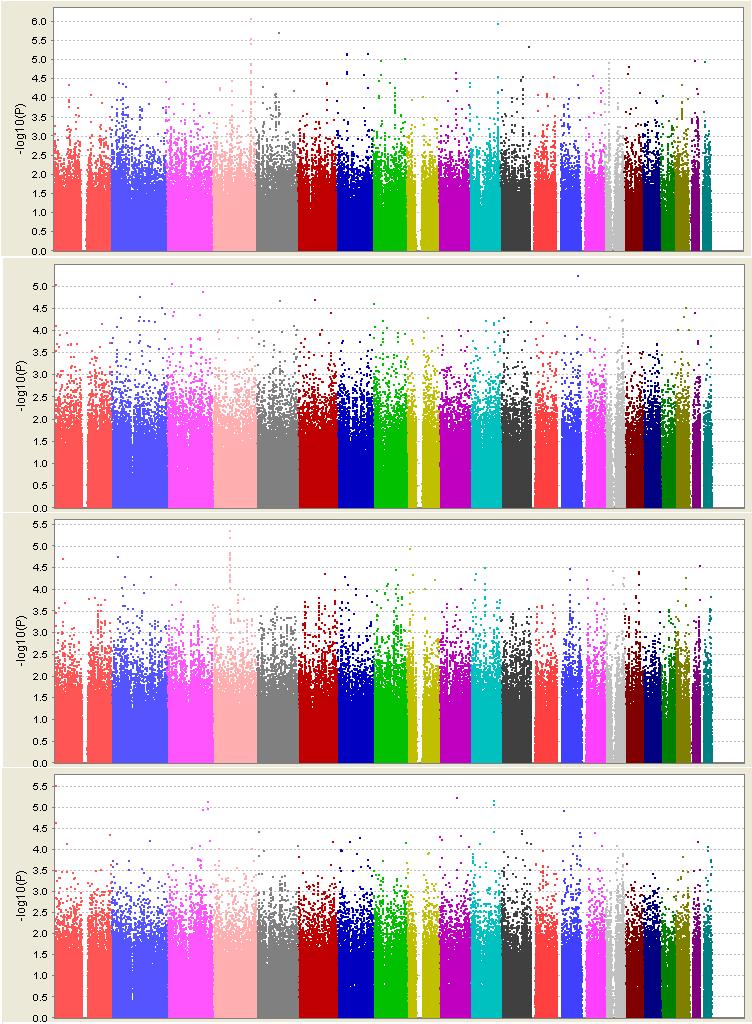


**(d)**

**(c)**

**(b)**

**(a)**

**
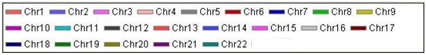
**

**Figure S2** **Manhattan plots of each genome-wide association analysis.** (a) FS-EA, (b) FS-AA, (c) CC-EA, (d) CC-AA. FS, factor score analysis; CC, case-control analysis; EA, European Americans; AA, African Americans. The –log_10_(p-value) is plotted according to its physical position on successive chromosome.


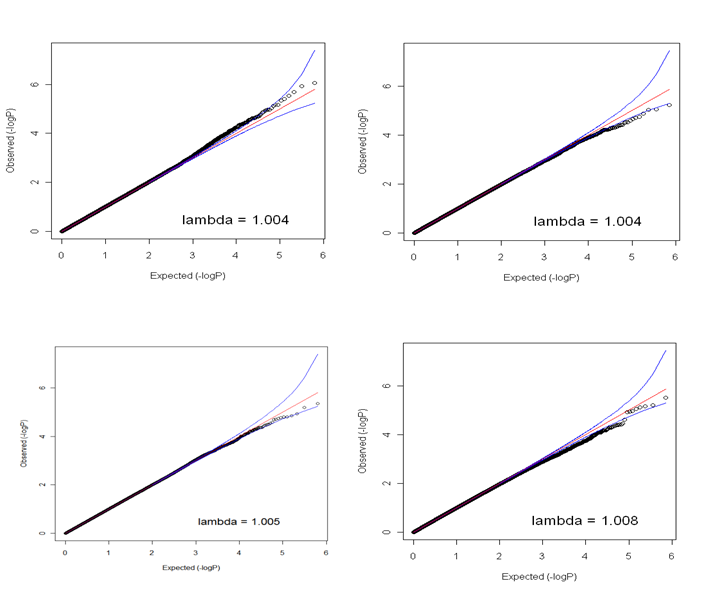


**(d)**

**(c)**

**(b)**

**(a)**

Expected (-logP)

Expected (-logP)

Expected (-logP)

Expected (-logP)

**Figure S3 Quantile-quantile (QQ) plots of gene-based genome-wide association analysis.** (a) FS-EA, (b) FS-AA, (c) CC-EA , (d) CC-AA. FS, factor score analysis; CC, case-control analysis; EA, European Americans; AA, African Americans. The observed –log_10_(p-value) are plotted against the expected –log_10_(p-value) under no association (straight red line) The 95% confidence interval of expected values is indicated in each figure (upper and lower blue line).
